# Etiologic Agents of Bacterial Sepsis and Their Antibiotic Susceptibility Patterns among Patients Living with Human Immunodeficiency Virus at Gondar University Teaching Hospital, Northwest Ethiopia

**DOI:** 10.1155/2016/5371875

**Published:** 2016-05-23

**Authors:** Gelila Alebachew, Brhanu Teka, Mengistu Endris, Yitayal Shiferaw, Belay Tessema

**Affiliations:** ^1^Department of Medical Microbiology, School of Biomedical and Laboratory Sciences, College of Medicine and Health Sciences, University of Gondar, Gondar, Ethiopia; ^2^Department of Microbiology, Immunology and Parasitology, Addis Ababa University, Addis Ababa, Ethiopia; ^3^Department of Medical Biotechnology, School of Medicine, Flinders University, Bedford Park, SA 5042, Australia

## Abstract

*Background*. Bacterial sepsis is a major cause of illness in human immunodeficiency virus infected patients. There is scarce evidence about sepsis among HIV patients in Ethiopia. This study aimed to determine the etiologic agents of bacterial sepsis and their antibiotic susceptibility patterns among HIV infected patients.* Methods*. A cross-sectional study was carried out from March 1 to May 2, 2013. One hundred patients infected with HIV and suspected of having sepsis were included. Sociodemographic data were collected by interview and blood sample was aseptically collected from study participants. All blood cultures were incubated aerobically at 35°C and inspected daily for 7 days. The positive blood cultures were identified following the standard procedures and antimicrobial susceptibility testing was performed using disk diffusion technique. Data was entered by Epi-info version 3.5.1 and analysis was done using SPSS version 20.* Results*. Of the study participants, 31 (31%) confirmed bacterial sepsis. The major isolates were 13 (13%)* Staphylococcus aureus*, 8 (8%) coagulates negative staphylococci, and 3 (3%) viridans streptococci. Majority of the isolates, 25 (80.6%), were multidrug resistant to two or more antimicrobial agents.* Conclusions*. Bacterial sepsis was a major cause of admission for HIV infected patients predominated by* Staphylococcus aureus* and coagulase negative staphylococci species and most of the isolates were multidrug resistant.

## 1. Introduction

Bacterial sepsis constitutes a significant public health problem and represents an important cause of morbidity and mortality in HIV/AIDS patients in the world. Epidemiologic studies have shown that 1% to 10% of the sepsis patients are individuals with HIV/AIDS in ICU [1]. Sepsis is systemic illness caused by microbial invasion of normally sterile parts of the body and commonly defined as the presence of infection with the systemic inflammatory response syndrome (SIRS); it is the presence of two or more of the following conditions: abnormal body temperature (<36°C (96.8°F) or >38°C (100.4°F)); heart rate (>90 beats/min); respiratory rate (>20 breaths/min); and white blood cell (WBC) count (<4000/mm^3^ or >12,000/mm^3^) [2].

The source of infection can be lung, genitourinary tract, gastrointestinal tract, skin or soft tissue, and so on [3]. Pathogenesis of bacteria due to the structural components of the Gram negative and Gram positive bacteria is responsible for initiating sepsis [4].

Sepsis can be caused by different types of bacteria. Gram negative bacteria are the most commonly isolated in septic patients and have lipopolysaccharide or endotoxin structure [5]. The percentage of patients with severe infections caused by Gram positive bacteria has increased in recent years, accounting for almost half of the incidents of septicemia and severe systemic infections [6, 7]. Gram positive bacteria have no endotoxin, but they cause sepsis indistinguishable from Gram negative bacteria by two distinct mechanisms, by releasing exotoxins that act as super antigens (e.g., staphylococci or streptococci) and cell wall components of Gram positive bacteria (both peptidoglycan and lipoteichoic acid) [7].

The clinical syndrome is caused by microorganism and its virulence factors with the host and its inflammatory response. Bacteria with special relevance for sepsis include Gram positive:* Streptococcus pneumonia*,* Streptococcus progenies*,* Streptococcus agalactiae*, and* Staphylococcus auras*; and Gram negative:* Neisseria meningitides*, enteric (*Escherichia coli*,* Klebsiella*,* Proteus*,* Enterobacter*,* Serratia*,* Citrobacter*, and* Salmonella*), and nonenteric (*Pseudomonas aeruginosa* and* Acinetobacter*) [5].

People living with HIV are at greater risk for sepsis because of immune suppression. In areas of high HIV prevalence, sepsis might contribute substantially to overall mortality [8, 9].

Emerging of multidrug resistant bacteria can reduce the management of bacterial infections. Bacterial infection to the HIV infected cases can mostly be caused by the multidrug resistant organism. Multidrug resistant organism is a bacterium that is resistant to more than one antibiotic. If bacteria are resistant to an antibiotic, certain drug treatments will not work [10].

Like in many other developing countries, bacterial sepsis and antibiotic resistant organism in Ethiopia are the most common disease for mortality and morbidity. But there is no evidence for bacterial sepsis among HIV/AIDS patients and their interaction. Hence, this study aimed to analyze the etiologic agents of bacterial sepsis and susceptibility pattern of bacterial isolates among HIV infected patients.

## 2. Materials and Methods

### 2.1. Study Area

The study was conducted at University of Gondar Teaching Hospital which is located in Gondar town, Northwest Ethiopia, 737 km away from Addis Ababa. The University Hospital was purposefully selected with the fact that it is one of the biggest tertiary level referral and teaching hospitals in the region visited by about six million people from the surrounding zones and nearby regions both for inpatient and for outpatient treatment. The hospital consists of ART pharmacy, ART laboratory, ART clinic, ICU with 16 beds, 13 wards with 327 beds, and outpatient departments.

### 2.2. Study Design and Population

Hospital based cross-sectional study was conducted among HIV/AIDS patients attending at ART clinic from March 1 to May 2, 2013.

### 2.3. Study Participants

All HIV/AIDS patients who were suspected of having sepsis and attended the University of Gondar Teaching Hospital during the study period were enrolled in the study.

### 2.4. Inclusion Criteria

All confirmed HIV patients that were suspected of having sepsis by clinicians were included in the study.

### 2.5. Exclusion Criteria

Patients receiving antibiotic treatment and neonates were excluded from the study.

### 2.6. Operational Definitions

- *Sepsis suspect* is a patient who fulfils definition of SIRS.
- *SIRS* is the presence of 2 or more of the following:
  - Temperature <36°C (96.8°F) (hypothermia) or >38°C (100.4°F).
  - Heart rate >90 beats/min (tachycardia).
  - Respiratory rate >20 breaths/min (tachypnea).
  - WBC count <4000/mm^3^ (leucopenia) or >12,000/mm^3^ (leukocytosis).

### 2.7. Sample Size and Sampling Technique

During the two-month study period, 100 study subjects were enrolled using convenient sampling technique from the study population visiting University of Gondar Teaching Hospital.

### 2.8. Data Collection and Laboratory Methods

A pretested, structured questionnaire was used to collect sociodemographic information, clinical signs, or symptoms by medical doctor. The pretest of the questionnaire was conducted in 10 patients at University of Gondar Teaching Hospital and pretest results were not included in the final results of the research but we used it to validate the questionnaire before conducting data collection.

#### 2.8.1. Sample Collection and Handling

Blood samples were collected from patients before antibiotic therapy by using venipuncture technique. Careful skin cleaning using tincture of iodine and 70% alcohol was done before drawing blood to prevent contamination. Ten mL of blood sample from adults and 2 mL of blood sample from children were aseptically collected following the request of the clinician. Then, it was inoculated into a blood culture bottle each containing 45 mL of tryptone soy broth (TSB) (Oxoid) [11].

#### 2.8.2. Culture and Identification

Blood cultures were incubated aerobically at 35°C and inspected daily for 7 days for the presence of visible microbial growth by observing any one of the following: turbidity, haemolysis, gas production, and coagulation of broth. For blood cultures that showed signs of microbial growth, subcultures were done onto blood, chocolate, and MacConkey agar (Oxoid). The blood and MacConkey agar plates were incubated in aerobic and chocolate agar in microaerophilic atmosphere using a candle jar at 35°C for 24–48 hours [11].

All positive blood cultures were identified by their characteristic appearance on their respective media and Gram staining reaction and confirmed by the pattern of biochemical reactions using the standard method. Gram negative bacteria were identified by indole production, citrate utilization, motility, urease, oxidase, gas production, hydrogen sulfide production, carbohydrate fermentation, and lysine decarboxylases production tests. For Gram positive bacteria: for staphylococci, coagulase, catalase test, and manifold salt agar were done and, for streptococci, bacitracin and opt chin susceptibility tests were done. Blood culture broth which shows no microbial growth within 7 days was reported as culture negative, only after results were subcultured on blood, MacConkey, and chocolate agar [11].

#### 2.8.3. Antimicrobial Susceptibility Testing

Antimicrobial susceptibility testing was performed for all isolates according to the criteria of the Clinical and Laboratory Standard Institute (CLSI) by disk diffusion method [12].

From a pure culture 3–5 selected colonies of bacteria were taken and transferred to a tube containing 5 mL of tryptone soy broth and mixed gently to a homogenous suspension and incubated at 35°C until the turbidity of the suspension becomes adjusted to a McFarland 0.5. A; sterile cotton swab was used and the excess suspension was removed by gentle rotation of the swab against the surface of the tube. The swab was then used to distribute the bacteria evenly over the entire surface of Mueller-Hinton agar (Oxoid) and for fastidious organisms' sensitivity was done on blood and chocolate agar plates [12].

The inoculated plates were left at room temperature to dry for 3–5 minutes and a set of 14 antibiotic discs (Oxoid) were placed 24 mm apart from each other on the surface of a Mueller-Hinton plate. Each disk was pressed down to ensure complete contact with the agar surface. The drugs for disc diffusion testing were in the following concentrations: ampicillin (10 *μ*g), amoxicillin-clavulanic acid (30 *μ*g), ceftriaxone (30 *μ*g), ciprofloxacin (5 *μ*g), chloramphenicol (30 *μ*g), sulfamethoxazole/trimethoprim (25 *μ*g), nitrofurantoin (50 *μ*g), erythromycin (15 *μ*g), gentamicin (10 *μ*g), methicillin (5 *μ*g), oxacillin (1 *μ*g), penicillin (10 IU), tetracycline (30 *μ*g), and vancomycin (30 *μ*g). Penicillin, vancomycin, erythromycin, oxacillin, and methicillin were tested only for Gram positive bacteria. The plates were then incubated at 35°C for 24–48 hours. Diameters of the zone of inhibition around the disc were measured to the nearest millimeter using the ruler, and the isolates were classified as sensitive and resistant according to the standardized table supplied by the CLSI [12].

#### 2.8.4. Quality Control

Standard reference strains of* Escherichia coli* (American Type Culture Collection- (ATCC-) 25922),* Staphylococcus aureus* (ATCC-25923), and* Pseudomonas aeruginosa* (ATCC-27853) were used as a quality control throughout the study for culture and antimicrobial susceptibility testing.

#### 2.8.5. Data Analysis and Interpretation

Data entry was done by Epi-info version 3.5.1 and analysis was done using statistical package for social science (SPSS) software version 20. Descriptive statistical methods were employed to describe magnitude and percentage of sepsis. Chi-square and odds ratio tests with 95% confidence interval were used to determine presence and strength of association. In all cases *P* value less than 0.05 was considered as statistically significant.

#### 2.8.6. Ethical Consideration

This study was approved by the Research and Ethical Committee of the School of Biomedical and Laboratory Sciences, College of Medicine and Health Sciences, University of Gondar. Participation was voluntary and informed written consent was obtained from each study participant/guardian. The laboratory result was sent to physicians for appropriate treatment of the study participants. All information about the patients was kept confidential.

## 3. Results

One hundred HIV positive patients suspected of having sepsis were included in this study. Out of this, 39 (39%) were males and 61 (61%) were females. The age range of participants was 5–53 years with mean and SD 33.2 ± 9.8. There was no significant association between the sociodemographic characteristics of participants and bacterial sepsis (Table 1).

Thirty-one isolates were obtained from blood samples of 31 HIV infected patients suspected of having sepsis. Gram positive isolates were predominantly recovered from the blood cultures, that is, 26 (83.4%) (Table 2).* S. aureus* 13 (42%) was the leading bacterial isolate followed by coagulase negative staphylococci species 8 (25.8%) in this study (Table 2). Five different Gram negative bacteria, namely,* E. coli*,* K. ozaenae*,* H. influenzae*,* P. aeruginosa,* and* Serratia* species, were also isolated from HIV infected patients.* P. aeruginosa* isolates were from a more extended (4 months) hospitalized child found in critical condition in the pediatrics ward.

### 3.1. Susceptibility Pattern of the Isolates

The antibiotic resistance patterns of the isolates to different antibiotics are described in Table 3. Ciprofloxacin, gentamicin, chloramphenicol, and oxacillin were active against >75% of Gram positive organisms. Amoxicillin-clavulanic acid, ceftriaxone, nitrofurantoin, and vancomycin were active against 100% of Gram positive isolates. Ciprofloxacin and nitrofurantoin are active against 100% of Gram negative isolates (Table 3).

The most common isolates,* S. aureus* and CONS, were resistant mainly to ampicillin, methicillin, penicillin, and tetracycline. Five* S. aureus* isolates were resistant to methicillin while there was no vancomycin resistant* S. aureus* in this study (Table 3).

All Gram negative isolates (5 in number) were resistant to cotrimoxazole and most were resistant to ampicillin 4 (80%), tetracycline 4 (80%), amoxicillin-clavulanic acid 4 (80%), and chloramphenicol 2 (40%). The majority of Gram positive isolates 17 (65.3%) were resistant to cotrimoxazole (Table 3).

All the five Gram negative isolates were also resistant to cotrimoxazole. The isolated* P. aeruginosa* was resistant to ampicillin, sulfamethoxazole, amoxicillin-clavulanic acid, ceftriaxone, gentamicin, tetracycline, and chloramphenicol except that of ciprofloxacin and nitrofurantoin (Table 3).

Majority of the isolates, 25 (80.6%), were multidrug resistant, to two or more antimicrobial agents (Table 4). The antibiogram of the two common bacterial isolates* S. aureus* and CONS species was presented (Table 5).

## 4. Discussion

From this study, bacterial sepsis is one of the important public health problems among HIV/AIDS patients (31%). But this frequency was lower than previous reports from China among surgically operated HIV/AIDS patients (41%) [13] and Africa among HIV infected children (53%) [14]. The magnitude difference might be due to the difference in the study participants in which most (94%) were adults (Table 1). In addition, lesser magnitude of bacterial sepsis among HIV infected patients than surgically operated patients might also be explained by the susceptibility of the latter group for local bacterial infections that lead to bacterial sepsis.

In the present study, Gram positive sepsis (83.4%) was more common than Gram negative sepsis (16.6%) (Table 2). This may be due to the fact that the percentage of patients with severe infections caused by Gram positive bacteria has increased in recent years, accounting for almost half of the incidents of septicemia and severe systemic infections [6, 7].

Of the 13* S. aureus* isolates, five (38.5%) were MRSA. Methicillin resistant* Staphylococcus aureus* were also reported in different studies among HIV infected patients with the prevalence of 43.5% MRSA bacteraemia [15] and 10.3% had MRSA colonization [16]. These results showed that MRSA are the common isolates among HIV infected patients. The second most common isolates were CONS, 8 (25.8%). A similar study also reported that CONS are the major cause of bacterial infection in HIV/AIDS patients (58%) [17].

The antibiotic susceptibility test results revealed that most isolates 22 (70.9%) were resistant for most antibiotics tested. Gram positive and Gram negative isolates showed 65.3% and 100% resistance for cotrimoxazole, respectively (Table 3). The reason for this high resistant isolates among HIV/AIDS patients might be multifactorial. Inappropriate use of the antibiotic by patients might be one possible reason for the high resistance since cotrimoxazole has been given as prophylaxis for HIV/AIDS patients. This action might also enhance the resistance to the antimicrobial agent. It is also very difficult to generalize with this sample size about the cotrimoxazole resistance among isolates of HIV/AIDS patients. Large scale comparative study might answer the question. From previous retrospective cohort study among HIV infected patients, most of the isolates resist cotrimoxazole widespread use for prophylaxis may exacerbate antimicrobial resistance [14].

Gram positive bacteria also showed good sensitivity patterns to amoxicillin-clavulanic acid, ceftriaxone, nitrofurantoin, and vancomycin.

Gram negative bacteria showed good sensitivity pattern only for ciprofloxacin and nitrofurantoin (Table 3). This might be due to the use of this specific antibiotic which is not widespread.

One* P. aeruginosa* isolate was from a more extended (4 months) hospitalized child found in critical condition in the pediatrics ward.* P. aeruginosa* have also been more frequently recognized as nosocomial bacteraemia in HIV infected patients [17].

## 5. Conclusions

The prevalence of culture that confirmed bacterial sepsis among HIV infected patients was high in this study (31%) and the two most common causative agents for bacterial sepsis are* S. aureus* and CONS. Amoxicillin-clavulanic acid, ceftriaxone, nitrofurantoin, and vancomycin were 100% active against Gram positive bacteria. Ciprofloxacin and nitrofurantoin were 100% active against Gram negative bacteria. Most of the isolates were multidrug resistant including cotrimoxazole.

## 6. Recommendations

Based on the findings of this study, we recommend that blood culture should be done for HIV infected patients suspected of having sepsis. Amoxicillin-clavulanic acid, ceftriaxone, nitrofurantoin, and vancomycin can be used against Gram positives if the laboratory setups are incomplete. Continuous antimicrobial susceptibility surveillance shall be implemented in the study area with large scale study among HIV/AIDS patients focusing on cotrimoxazole.

## Figures and Tables

**Table 1:** Sociodemographic characteristics of HIV/AIDS patients suspected of having sepsis admitted to University of Gondar Teaching Hospital with their bacterial sepsis status, March 1, 2013, to May 2, 2013.

Characteristics	Confirmed bacterial sepsis		Total number (%)	*χ* ^2^	*P* value
	Yes number (%)	No number (%)			
Sex					
Male	12 (30.8)	27 (69.2)	39 (39)	0.00	0.96
Female	19 (31.1)	42 (68.9)	61 (61)		
Age (years)					
<18	2 (33.3)	4 (66.7)	6 (6)	0.02	0.99
18+	29 (30.9)	65 (69.1)	94 (94)		
Residence					
Urban	16 (36.4)	28 (63.6)	44 (44)	1.06	0.30
Rural	15 (26.8)	41 (73.2)	56 (56)		
Educational status					
Literate	17 (32.7)	35 (67.3)	52 (52)	0.15	0.70
Illiterate	14 (29.2)	34 (70.8)	48 (48)		
Marital status					
Single	8 (27.6)	21 (72.4)	29 (29)	1.28	0.73
Married	18 (30.0)	42 (70.0)	60 (60)		
Divorced	4 (44.4)	5 (55.6)	9 (9)		
Widowed	1 (50.0)	1 (50.0)	2 (2)		
Occupation					
Farmer	5 (29.4)	12 (70.6)	17 (17)	2.49	0.96
House servant	4 (28.6)	10 (71.4)	14 (14)		
House wife	5 (23.8)	16 (76.2)	21 (21)		
Daily labor	5 (50.0)	5 (50.0)	10 (10)		
Merchant	2 (28.6)	5 (71.4)	7 (7)		
Employer (Gov't)	5 (35.7)	9 (64.3)	14 (14)		
Jobless	3 (30.3)	7 (70.0)	10 (10)		
Driver	1 (25.0)	3 (75.0)	4 (4)		
Others^*∗*^	1 (33.3)	2 (66.7)	3 (3)		

*Total*	*31 *(*31*)	*69 *(*69*)	*100 *(*100*)		

*∗*: builder, waiter, and working as hair stylist, *χ* ^2^ = chi-squares.

**Table 2:** Frequency of bacterial etiologies among HIV/AIDS patients in the University of Gondar Teaching Hospital from March 1, 2013, to May 2, 2013.

Etiologic agent	Frequency	Percent
*Gram positive *((*n = 26* (*26%*))		
*S. aureus*	13	42
CONS	8	25.8
Viridans streptococci	3	9.7
*S. pyogenes*	2	6.5
*Gram negative* ((*n = 5*) (*5%*))		
*E. coli*	1	3.2
*K. ozaenae*	1	3.2
*H. influenzae*	1	3.2
*P. aeruginosa*	1	3.2
*Serratia* species	1	3.2

*Total *	31	100

CONS: coagulase negative *Staphylococcus*.

**Table 3:** Resistance pattern of bacterial isolates to commonly used antibiotics among HIV infected patients in the University of Gondar Teaching Hospital from March 1, 2013, to May 2, 2013.

Organism isolated	Antibiotic resistance patterns													
	SXT	AMC	CRO	CIP	E	CN	MET	P	TE	V	CAF	OXA	AMP	F
*Gram positive*														
*S. aureus *(*n* = 13)	6 (46.2)	0 (0)	0 (0)	0 (0)	3 (23.1)	0 (0)	5 (38.5)	5 (38.5)	4 (30.8)	0 (0)	1 (7.7)	3 (23.1)	11 (84.6)	0 (0)
CONS (*n* = 8)	8 (100)	0 (0)	0 (0)	1 (12.5)	3 (37.5)	1 (12.5)	6 (75.0)	3 (37.5)	4 (50.0)	0 (0)	2 (25.0)	2 (25.0)	3 (37.5)	0 (0)
Viridans streptococci(*n* = 3)	1 (33.3)	0 (0)	0 (0)	0 (0)	0 (0)	2 (66.7)	1 (33.3)	0 (0)	0 (0)	0 (0)	0 (0)	1 (33.3)	1 (33.3)	0 (0)
*S. pyogenes *(*n* = 2)	2 (100)	0 (0)	0 (0)	0 (0)	1 (50)	0 (0)	1 (50)	1 (50)	0 (0)	0 (0)	0 (0)	0 (0)	2 (100)	0 (0)
Total (*n* = 26)	17 (65.3)	0 (0)	0 (0)	1 (3.8)	7 (26.9)	3 (11.5)	13 (0.5)	9 (34.6)	8 (30.7)	0 (0)	3 (11.5)	6 (23.0)	17 (65.3)	0 (0)

*Gram negative*														
*E. coli* (*n* = 1)	1 (100)	1 (100)	0 (0)	0 (0)	—	0 (0)	—	—	1 (100)	—	0 (0)	—	1 (100)	0 (0)
*K. ozaenae* (*n* = 1)	1 (100)	1 (100)	0 (0)	0 (0)	—	0 (0)	—	—	1 (100)	—	0 (0)	—	1 (100)	0 (0)
*H. influenzae* (*n* = 1)	1 (100)	0 (0)	0 (0)	0 (0)	—	0 (0)	—	—	0 (0)	—	0 (0)	—	0 (0)	0 (0)
*P. aeruginosa *(*n* = 1)	1 (100)	1 (100)	1 (100)	0 (0)	—	1 (100)	—	—	1 (100)	—	1 (100)	—	1 (100)	0 (0)
*Serratia* (*n* = 1)	1 (100)	1 (100)	0 (0)	0 (0)	—	0 (0)	—	—	1 (100)	—	1 (100)	—	1 (100)	0 (0)
*Total *(*n = 5*)	5 (100)	4 (80)	1 (20)	0 (0)	—	1 (20)	—	—	4 (80)	—	2 (40)	—	4 (80)	0 (0)

*Overall *(*n = 31*)	22 (70.9)	4 (12.9)	1 (3.2)	1 (3.2)	7 (26.9)	4 (12.9)	13 (0.5)	9 (34.6)	12 (38.7)	0 (0)	5 (16.1)	6 (23.0)	21(67.7)	0 (0)

AMP: ampicillin; SXT: sulfamethoxazole/trimethoprim; AMC: amoxicillin-clavulanic acid; CRO: ceftriaxone; CIP: ciprofloxacin; E: erythromycin; CN: gentamicin; MET: methicillin; P: penicillin; TE: tetracycline; V: vancomycin; CAF: chloramphenicol; OXA: oxacillin; F: nitrofurantoin; S: sensitive; I: intermediate; *R*: resistant; —: not used antibiotic for Gram negative.

**Table 4:** Antibiotic resistance profile of bacterial isolate among HIV infected patients in the University of Gondar Teaching Hospital from March 1, 2013, to May 2, 2013.

Bacterial isolate	*R*_0_ (%)	*R*_1_ (%)	*R*_2_ (%)	*R*_3_ (%)	*R*_4_ (%)	*R*_5_ and above (%)
*Gram positive*						
*S. aureus* (*n* = 13)	1 (7.6)	1 (7.6)	5 (38.4)	2 (15.3)	1 (7.6)	3 (23.0)
CONS (*n* = 8)	0 (0)	1 (12.5)	3 (37.5)	1 (12.5)	0 (0)	3 (37.5)
Viridans streptococci(*n* = 3)	0 (0)	2 (66.6)	0 (0)	0 (0)	1 (33.3)	0 (0)
*S. pyogenes *(*n* = 2)	0 (0)	0 (0)	0 (0)	1 (50)	0 (0)	1 (50)
*Total (26)*	1 (3.8)	4 (15.4)	8 (30.8)	4 (15.4)	2 (7.7)	7 (26.9)
*Gram negative*						
*E. coli* (*n* = 1)	0 (0)	0 (0)	0 (0)	0 (0)	1 (100)	0 (0)
*K. ozaenae* (*n* = 1)	0 (0)	0 (0)	0 (0)	0 (0)	1 (100)	0 (0)
*H. influenzae* (*n* = 1)	0 (0)	1 (100)	0 (0)	0 (0)	0 (0)	0 (0)
*P. aeruginosa *(*n* = 1)	0 (0)	0 (0)	0 (0)	0 (0)	0 (0)	1 (100)
*Serratia* (*n* = 1)	0 (0)	0 (0)	0 (0)	0 (0)	0 (0)	1 (100)
*Total *(*n = 5*)	0 (0)	1 (20)	0 (0)	0 (0)	2 (40)	2 (00)

*Overall *(*n = 31*)	1 (3.2)	5 (16.1)	8 (25.8)	4 (12.9)	4 (12.9)	9 (29.0)

*R*_0_: nonresistant; *R*_1_: resistant for one antibiotic; *R*_2_: resistant for two antibiotics; *R*_3_: resistant for three antibiotics; *R*_4_: resistant for four antibiotics; *R*_5_ and above: resistant for five and greater than five antibiotics.

**Table 5:** Multidrug resistance profile of frequently isolate bacteria among HIV infected patients in the University of Gondar Teaching Hospital from March 1, 2013, to May 2, 2013.

Bacterial species	Antimicrobial agents	Number of resistant isolates (%)
*S. aureus*	AMP and P	2 (15.3)
	AMP and SXT	2 (15.3)
	AMP and MET	1 (7.6)
	AMP, MET, and OXA	1 (7.6)
	AMP, SXT, and P	1 (7.6)
	AMP, SXT, E, and TE	1 (7.6)
	AMP, SXT, E, TE, and CAF	1 (7.6)
	AMP, SXT, MET, TE, P, and OXA	1 (7.6)
	AMP, P, E, TE, MET, and OXA	1 (7.6)

*CONS*	AMP and SXT	1 (12.5)
	SXT and E	1 (12.5)
	SXT and MET	1 (12.5)
	SXT, MET, and E	1 (12.5)
	SXT, MET, OXA, P, and TE	1 (12.5)
	AMP, MET, CAF, CIP, E, P, SXT, and TE	1 (12.5)
	AMP, MET, CAF, CN, E, OXA, P, SXT, and TE	1 (12.5)

AMP: ampicillin; SXT: sulfamethoxazole/trimethoprim; CIP: ciprofloxacin; E: erythromycin; CN: gentamicin; MET: methicillin; P: penicillin; TE: tetracycline; CAF: chloramphenicol; OXA: oxacillin; CONS: coagulase negative staphylococci.
